# Analysis of imported Chikungunya cases in China from 2006 to 2026

**DOI:** 10.3389/fmicb.2026.1856429

**Published:** 2026-05-29

**Authors:** Jiayi Wang, Wenxin Sun, Junxiang Zhao, Leiliang Zhang

**Affiliations:** 1 Department of Clinical Laboratory Medicine, The First Affiliated Hospital of Shandong First Medical University and Shandong Provincial Qianfoshan Hospital, Jinan, Shandong, China; 2 Department of Pathogen Biology, School of Clinical and Basic Medical Sciences, Shandong First Medical University and Shandong Academy of Medical Sciences, Jinan, Shandong, China

**Keywords:** Chikungunya fever, China, imported cases, public health, travel medicine

## Abstract

**Background:**

Chikungunya fever (CHIKF) has impacted 119 countries and regions globally. In 2025, a local outbreak occurred in Guangdong, China, driven by imported cases from Sri Lanka, resulting in over 25,000 diagnoses. This highlights CHIKF as a significant public health concern. Analyzing imported cases in China and discussing travel medicine and related preventive measures is essential.

**Methods:**

This study reviewed literature and reports on CHIKF published in international and Chinese databases, as well as government reports, from 2006 to March 1, 2026. Data related to CHIKF, including case numbers, virus types, source countries, and high-risk provinces, were collected, synthesized, and summarized using descriptive statistical methods. Phylogenetic tree analysis, temporal signal assessment, and mutation analysis were conducted on the Chikungunya virus (CHIKV) sequences sourced from China.

**Results:**

Between 2006 and 2026, China reported a total of 259 imported CHIKF cases, including those from Hong Kong, Macao, and Taiwan. These imported cases contributed to over 25,000 local transmission cases. Among confirmed cases, the East/Central/South African (ECSA) strain was the most prevalent (61.7%), followed by the Asian strain (37.4%). The primary countries of acquisition were Indonesia and Myanmar, with the Asian strain mostly detected in travelers returning from Indonesia, while the ECSA strain was found in travelers from South and Southeast Asia. The rate of evolution for Chinese cases mirrors that of global trends, and no new branches have emerged. The newly identified mutations in recent years exhibit high-frequency variations in the sequences, along with potential unidentified mutations that have appeared.

**Conclusion:**

Returnees from countries with high incidence rates of CHIKF significantly increase the risk of reintroducing the disease to China. An increased number of mutations may enhance the transmission of CHIKV. Therefore, it is crucial to enhance the dissemination of relevant prevention and control measures and expedite vaccine approval to mitigate the incidence of CHIKF among travelers and reduce viral transmission.

## 1 Introduction

Chikungunya virus (CHIKV) is an enveloped alphavirus belonging to the *Togaviridae* family, characterized by its single-stranded, positive-sense RNA genome (de Souza et al., 2025). The term “Chikungunya,” derived from the Maunganui language, translates to “bent” or “twisted,” which reflects the severe joint pain and stooped posture associated with the disease. CHIKV causes an acute febrile illness known as Chikungunya fever (CHIKF), characterized by fever, rash, and severe polyarthralgia (de Souza et al., 2025). The virus is primarily transmitted by the *Aedes aegypti* and *Aedes albopictus* mosquitoes (de Souza et al., 2025).

First identified in Tanzania in 1952 (Robinson, 1955), CHIKF has since spread to 119 countries and regions, affecting nearly 300,000 individuals worldwide by September 30, 2025, thereby establishing itself as a significant public health concern (WHO, 2025).

In China, CHIKF is not classified as a Class A, B, or C infectious disease, leading to the implementation of specialized case reporting and epidemic management methods. The Chinese government categorizes regions into four transmission risk levels (I, II, III, and IV), which can be dynamically adjusted based on changing epidemiological conditions. The Chikungunya Fever Control Technical Guidelines (2025 Edition) from the China Center for Disease Control and Prevention delineates the cases into three classifications: suspected, clinically diagnosed, and confirmed (CDC, 2025). These are further subdivided based on the source of acquisition into imported cases (from abroad and from other provinces) and local cases. This structured classification aids in effective epidemic management.

For imported cases, a strategy of “emergency vector control, ensuring case treatment, and isolated mosquito prevention” is adopted to avert local epidemics. Serum samples from suspected patients undergo testing for IgM and IgG antibodies via ELISA and nucleic acid detection by qPCR. Structural protein genes of the virus are amplified through RT-PCR and subsequently sequenced. Upon detection of CHIKF cases, medical institutions are mandated to report them online within 24 h, recording the information in the China Disease Prevention and Control Information System. Cases are then reviewed and validated based on laboratory results and epidemiological investigations. In instances of new, clustered, or outbreak situations, public health emergency reports are required to be filed within 2 h. During non-epidemic seasons, the focus for medical institutions is on treatment and mosquito prevention. In contrast, the epidemic season necessitates comprehensive epidemiological investigations and adult mosquito control measures.

The first isolation of CHIKV from bats in China occurred in 1986 (Zhang et al., 1989). The following year, the virus was isolated from fever patients in China for the first time (Cai et al., 2022). The first imported cases on the Chinese mainland were recorded in 2008 (Zheng et al., 2010), and a significant community outbreak linked to imported cases occurred in Dongguan, Guangdong Province, in 2010 (Qiaoli et al., 2012). In 2025, Guangdong Province experienced the largest CHIKF outbreak to date, which drew considerable attention from various sectors of society (Wang et al., 2025).

Despite comprehensive mosquito control measures and epidemic prevention efforts implemented by the Chinese government, the country continues to face ongoing risks associated with imported cases stemming from international travel and freight. The introduction of policies, such as the 240-h visa-free transit policy, has resulted in increased international interactions. In 2025, the volume of inbound and outbound travelers in China reached 697 million, with many entering from regions where CHIKF is prevalent, such as Southeast Asia and South Asia. While rapid testing equipment is available at entry ports, investigative means are limited, and diagnostic capabilities at the grassroots level vary significantly, which can facilitate local transmission (Han, 2020).

This study aims to describe the epidemic trajectory of CHIKV in China, assess the risk of CHIKV importation, and emphasize the necessity of enhancing access to travel medical care services for the majority of travelers, providing relevant recommendations in light of these findings.

## 2 Methods

### 2.1 Data sources and eligibility criteria

This study utilized both primary and aggregated data extracted from published articles. We systematically retrieved articles published in various databases, including Chinese-specific databases, focusing on the importation of CHIKV in China over the years, in addition to grey literature. Keyword searches were conducted using terms such as “China,” “Chikungunya” (or “CHIKV”), and “imported” across three databases: China National Knowledge Infrastructure (CNKI), Wanfang Data, and PubMed. The grey literature included government reports from the Hong Kong Special Administrative Region Centre for Health Protection^1^ and the Chinese Center for Disease Control and Prevention.^2^ The data from grey literature for Macao was obtained from Communicable Disease Prevention and Control Division, Center for Disease Control and Prevention, Health Bureau, Government of Macao Special Administrative Region.

Articles were included in the data extraction and synthesis if they met all the following inclusion criteria: (i) peer-reviewed articles or official government reports on CHIKF in China; (ii) data on CHIKF sourced from the Chinese Center for Disease Control and Prevention or other formal governmental institutions; and (iii) articles or reports that provide data on CHIKF in one or multiple regions over a defined period.

Articles were excluded if they fell into any of the following categories: (i) documents or articles providing advice on the diagnosis and treatment of CHIKF or on measures for prevention and control; (ii) articles or reports discussing CHIKF in other countries, except for cases related to China; (iii) relevant guidelines and expert consensus; and (iv) research and reports focused on transmission vectors or the development of new drugs.

### 2.2 Data extraction and synthesis

Data related to cases—including suspected cases, clinically diagnosed cases, and laboratory-confirmed cases—were extracted and recorded in an Excel spreadsheet. The type of cases (imported cases from abroad), virus strains, countries of acquisition, high-risk provinces for introduction (reported cases), and reasons and times of entry were all included.

The articles included provided information on imported cases since the beginning of importation history in China, including details on the virus strains (though some did not specify) and countries of acquisition. We summarized the data by counting the number of imported cases in high-risk provinces and acquisition countries. We analyzed the data related to three CHIKV strains: the Asian genotype, the West African genotype (WA), and the East/Central/Southern African genotype (ECSA) (Pialoux et al., 2007). Each strain has slightly different prevalence areas and preferred vectors (Vega-Rúa et al., 2014). Additionally, we considered ecological factors affecting the vectors (Yang et al., 2025) and changes brought about by national policies.

### 2.3 Statistical analysis

The extracted data were summarized through descriptive analysis and presented in tables, charts, and maps. Statistical analyses and charting were conducted using GraphPad Prism 10.5.0 (GraphPad Software, San Diego, CA, USA), while maps were created using ArcGIS software 10.8 (ESRI, Redlands, California).

### 2.4 CHIKV sequences

We selected 72 CHIKV genomic sequences from GenBank, all sampled in China between 2006 and 2026, and compiled them into a final dataset for bioinformatics analysis.

### 2.5 Phylogenetic tree construction

Sequences were aligned using MAFFT (Katoh et al., 2002). A maximum likelihood (ML) phylogenetic tree was constructed with IQ-TREE version 3.0.1 (Minh et al., 2020) using the optimal nucleotide substitution model TIM2 + F + I + R3, as determined by the Bayesian Information Criterion. Branch support was assessed using 1,000 ultrafast bootstrap replicates, and all final visualizations were generated with R version 4.5.2.

### 2.6 Temporal signal analysis

Temporal signal was evaluated using root-to-tip regression in TempEst v1.5.3 (Rambaut et al., 2016), and visualizations were created with GraphPad Prism 10.5.0 (GraphPad Software, San Diego, CA, USA).

### 2.7 Mutation analysis

Mutation analysis was performed on the CHIKV sequences from China, covering the years 2006 to 2026. The amino acid sequences of the structural proteins (capsid-E1) and the non-structural polyprotein (nsP1-nsP4) were aligned and compared to the CHIKV reference sequence (AF369024.2) to identify amino acid substitutions. Visualization was conducted using R version 4.5.2 and Jalview version 2.11.5.1.

## 3 Results

### 3.1 Total CHIKV imported cases and trends

**Figure 1:**
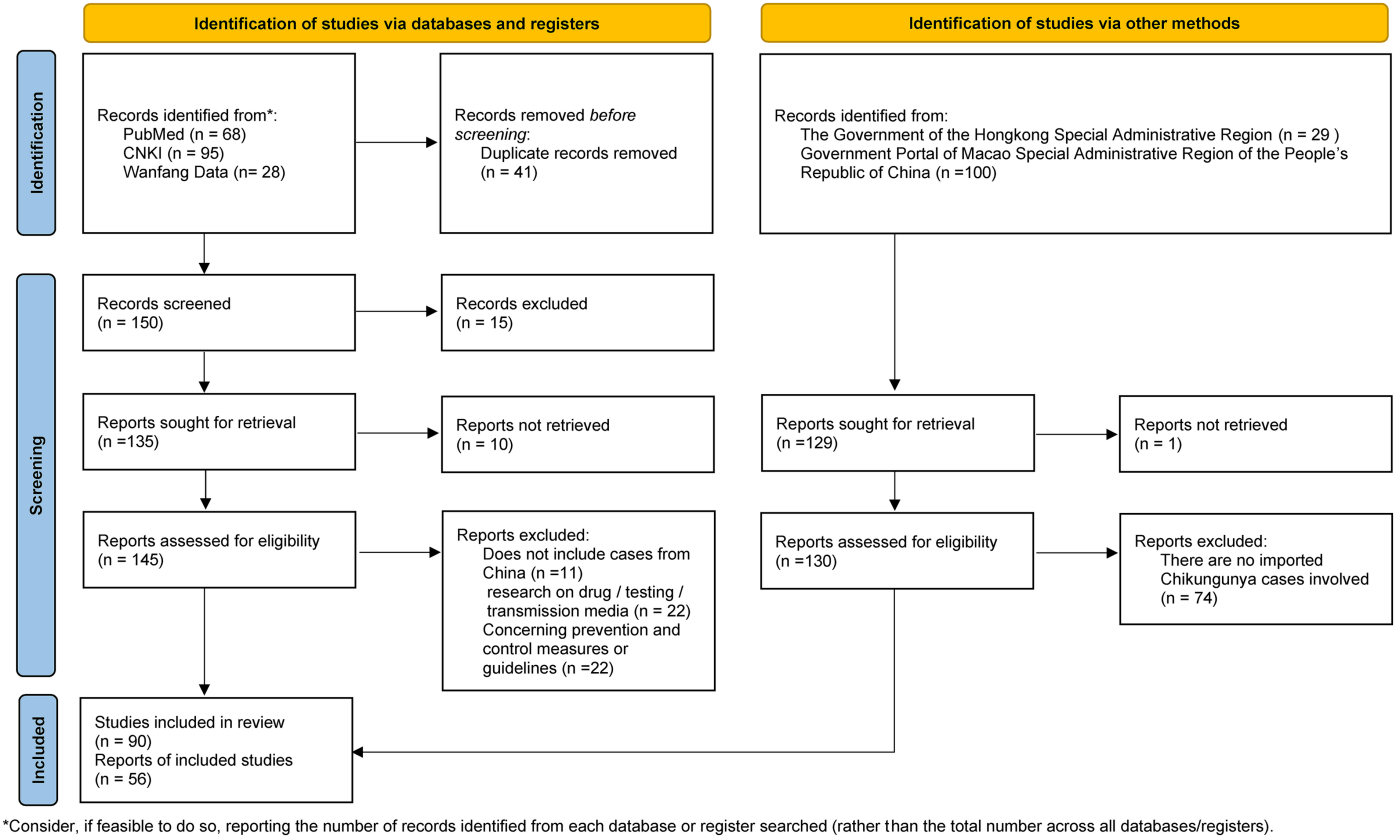
PRISMA 2020 flow diagram for new systematic reviews which included searches of databases, registers, and other sources.

A total of 201 articles and 130 government reports were retrieved. Among them, 90 articles and 56 government reports met the inclusion criteria and were included in the statistical analysis (Figure 1). All these articles and reports provided detailed information on imported cases of CHIKF in China, including the country of acquisition, time of importation, and, in some cases, the virus strains and reasons for entry into China. Virus strain information for the imported cases was primarily derived from genetic sequencing results.

The majority of acquisition countries were located in South and Southeast Asia, with sporadic reports also emerging from the Americas and Africa. High-risk provinces for imported cases predominantly included southeastern coastal areas and regions with frequent international economic interactions or geographical accessibility. Cross-provincial transmission has further contributed to the epidemic development, with 97 cumulative cases imported from outside the province. In recent years, there have been over 25,000 local confirmed cases, as reported by the China CDC.

### 3.2 CHIKV strains in imported cases

**Figure 2:**
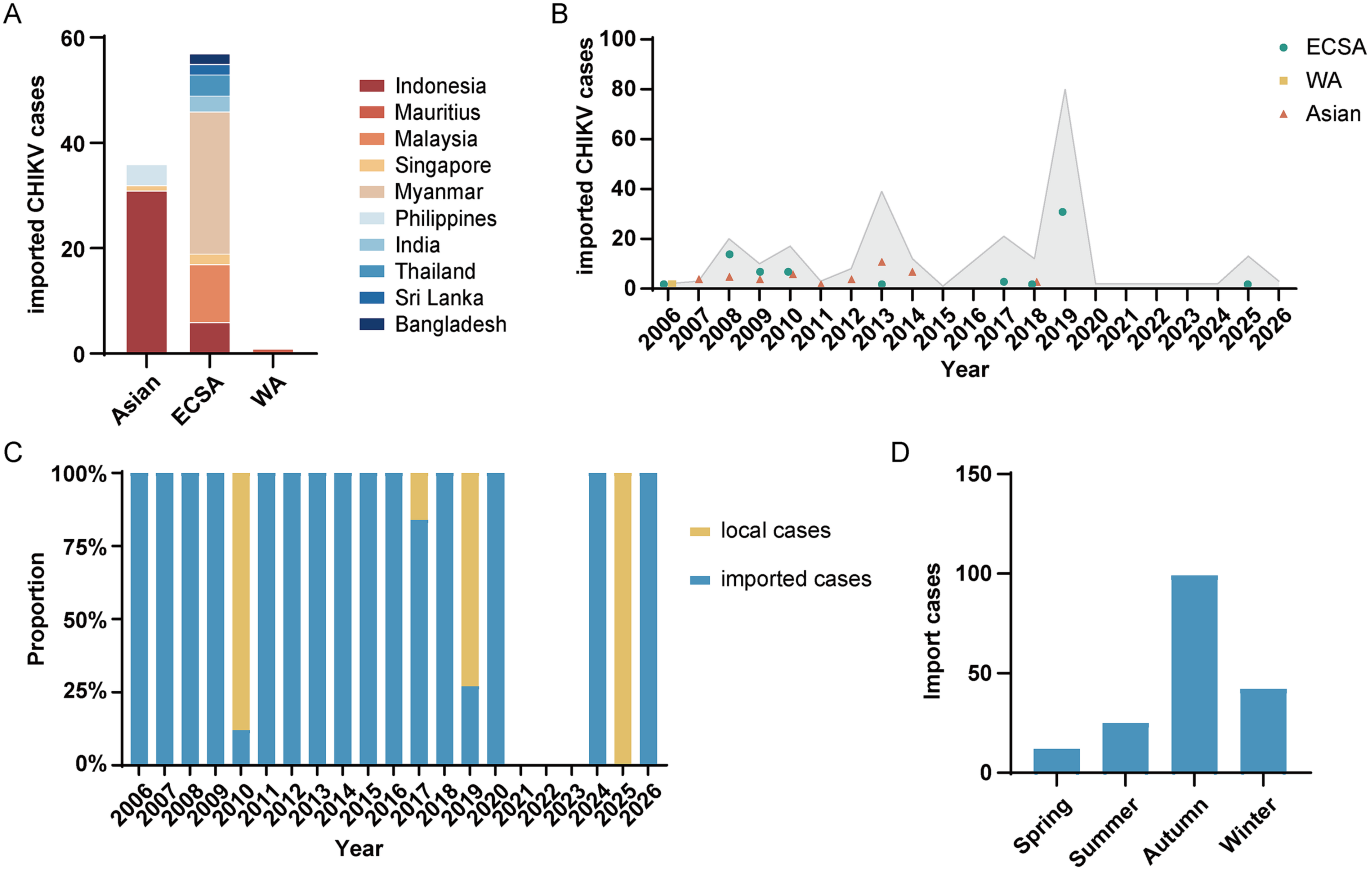
Epidemiological overview of CHIKV strains and cases. **(A)** Distribution of Asian, ECSA, and WA virus strains in the countries of acquisition. **(B)** Statistical analysis of the number and strains of imported cases by year. **(C)** The proportion of total confirmed cases (including both local and imported cases) from 2006 to March 1, 2026. **(D)** Distribution of imported cases in China during different seasons (excluding Hong Kong and Taiwan).

The ECSA strain was found to be the most prevalent among the imported cases, accounting for 61.7% of the total. The Asian strain followed, comprising 37.4% (Figure 2A). ECSA has consistently dominated from 2006 to 2026 (Figure 2B). In contrast, WA strain had an extremely low occurrence rate, with only one reported imported case in China (Figures 2A,B and Table 1).

**Table 1:** Distribution of CHIKV among imported CHIKF cases in China from 2006 to March 2, 2026, based on selected literature.

Year	Imported cases	CHIKV		
		ECSA	Asian	WA
2006	2	1	0	1
2007	3	0	3	0
2008	20	13	4	N/A
2009	10	6	3	N/A
2010	17	6	5	N/A
2011	3	N/A	1	N/A
2012	8	N/A	3	N/A
2013	39	1	10	N/A
2014	12	N/A	6	N/A
2015	1	N/A	N/A	N/A
2016	11	N/A	N/A	N/A
2017	21	2	N/A	N/A
2018	12	1	2	N/A
2019	80	30	N/A	N/A
2020	2	N/A	N/A	N/A
2021	N/A	N/A	N/A	N/A
2022	N/A	N/A	N/A	N/A
2023	N/A	N/A	N/A	N/A
2024	2	N/A	N/A	N/A
2025	13	1	N/A	N/A
2026	3	N/A	N/A	N/A

N/A data were unavailable.

### 3.3 Imported CHIKF cases and local pandemics

There have been three major local outbreaks of CHIKV in China. In addition to the 2025 Guangdong epidemic, the first recorded community outbreak occurred in Dongguan, Guangdong, in 2010 (Qiaoli et al., 2012), followed by an outbreak in Ruili, Yunnan, in 2019 (Figure 2C) (Liu et al., 2022). Each of these outbreaks involved a significant number of imported cases, primarily of the ECSA strain (Figure 2B). Summer and autumn are the seasons when imported cases of CHIKV are most likely to occur (Figure 2D).

### 3.4 Main countries of acquisition

**Figure 3:**
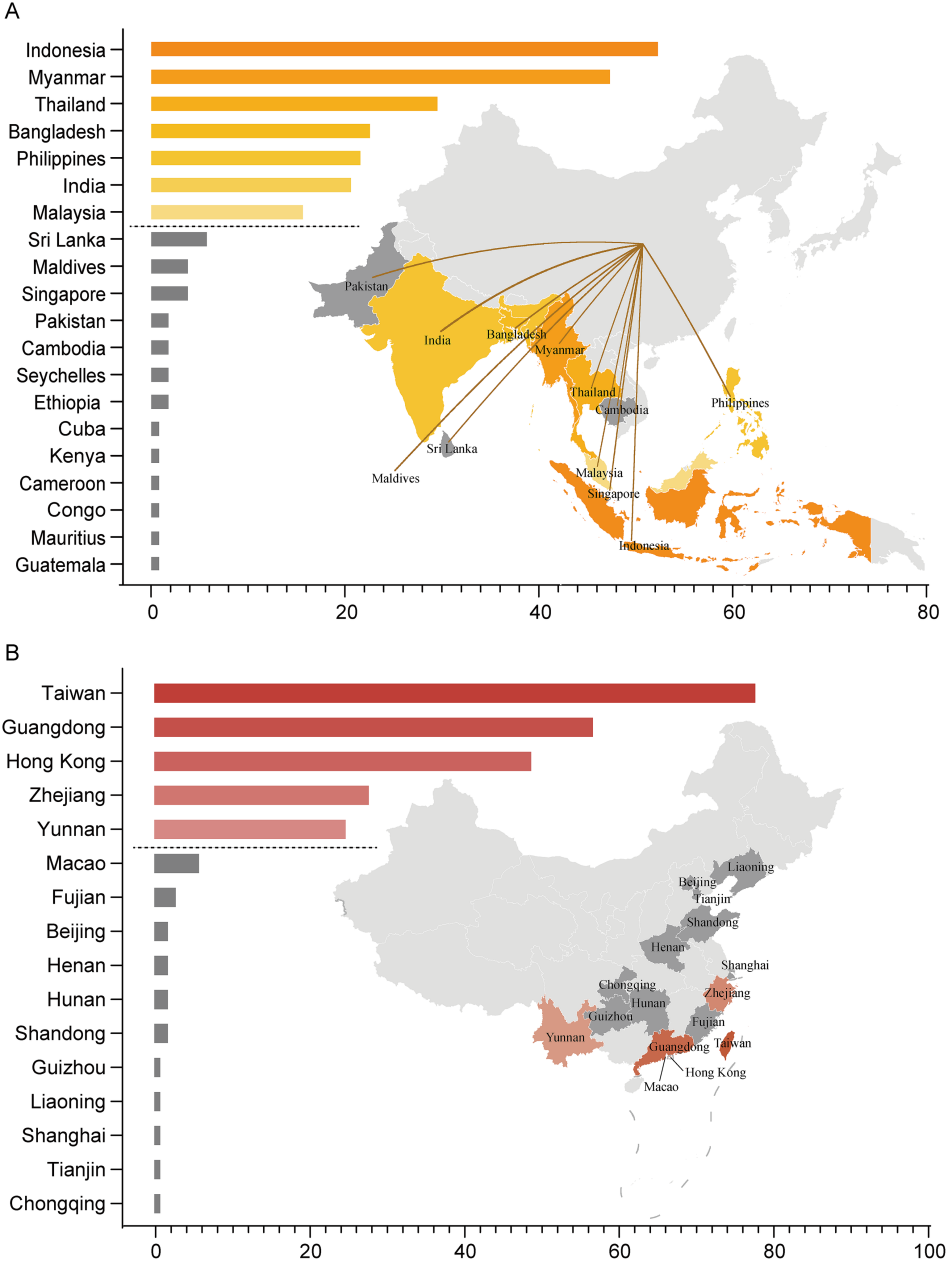
Distribution of imported cases of CHIKV. **(A)** Main countries of acquisition of imported CHIKF cases from South and Southeast Asia into China from 2006 to 2026. **(B)** Provinces in China reporting CHIKF cases from 2006 to 2026 (statistics for the Taiwan region are available up to 2020).

The primary countries of acquisition were located in South and Southeast Asia, with Indonesia ranking first (*n =* 53), followed by Myanmar (*n =* 48) (Figure 3A). Countries in South and Southeast Asia accounted for 95.6% of the total cases. According to the current data, the majority of imported cases in China are of the ECSA strain (*n =* 61) and the Asian type (*n =* 37). Among the Asian type, 83.8% of the imported cases originated from Indonesia, while the ECSA strain was predominantly sourced from countries in South Asia and Southeast Asia (98.4%) (Figure 2B and Table 2). The main reason for importation was returning home after international travel, accounting for 64.1%, followed by work-related travel (23.1%) and visitation (12.8%).

**Table 2:** Distribution of CHIKV among imported CHIKF cases by country of acquisition from 2006 to March 2, 2026, based on selected literature.

	ECSA	Asian	WA
Indonesia	6	31	1
Philippines	N/D	4	N/D
Singapore	2	1	N/D
India	3	N/D	N/D
Thailand	4	N/D	N/D
Sri Lanka	2	N/D	N/D
Myanmar	27	N/D	N/D
Bangladesh	2	N/D	N/D
Malaysia	11	N/D	N/D
Mauritius	N/D	N/D	N/D

Only cases with available genotypes are counted, not all imported cases. N/D data were not determined.

### 3.5 Provinces with higher risk of re-introduction and further transmission

Imported cases of CHIKF were primarily reported in Taiwan (*n =* 78), Guangdong (*n =* 57), Hong Kong (*n =* 49), Zhejiang (*n =* 28), and Yunnan (*n =* 25). These provinces are situated along the southeastern coast of China or border countries with high incidences of CHIKF. Additional imported cases were also identified in other cities (Figure 3B).

### 3.6 Phylogenetic analysis of the CHIKV sequences sampled from China

**Figure 4:**
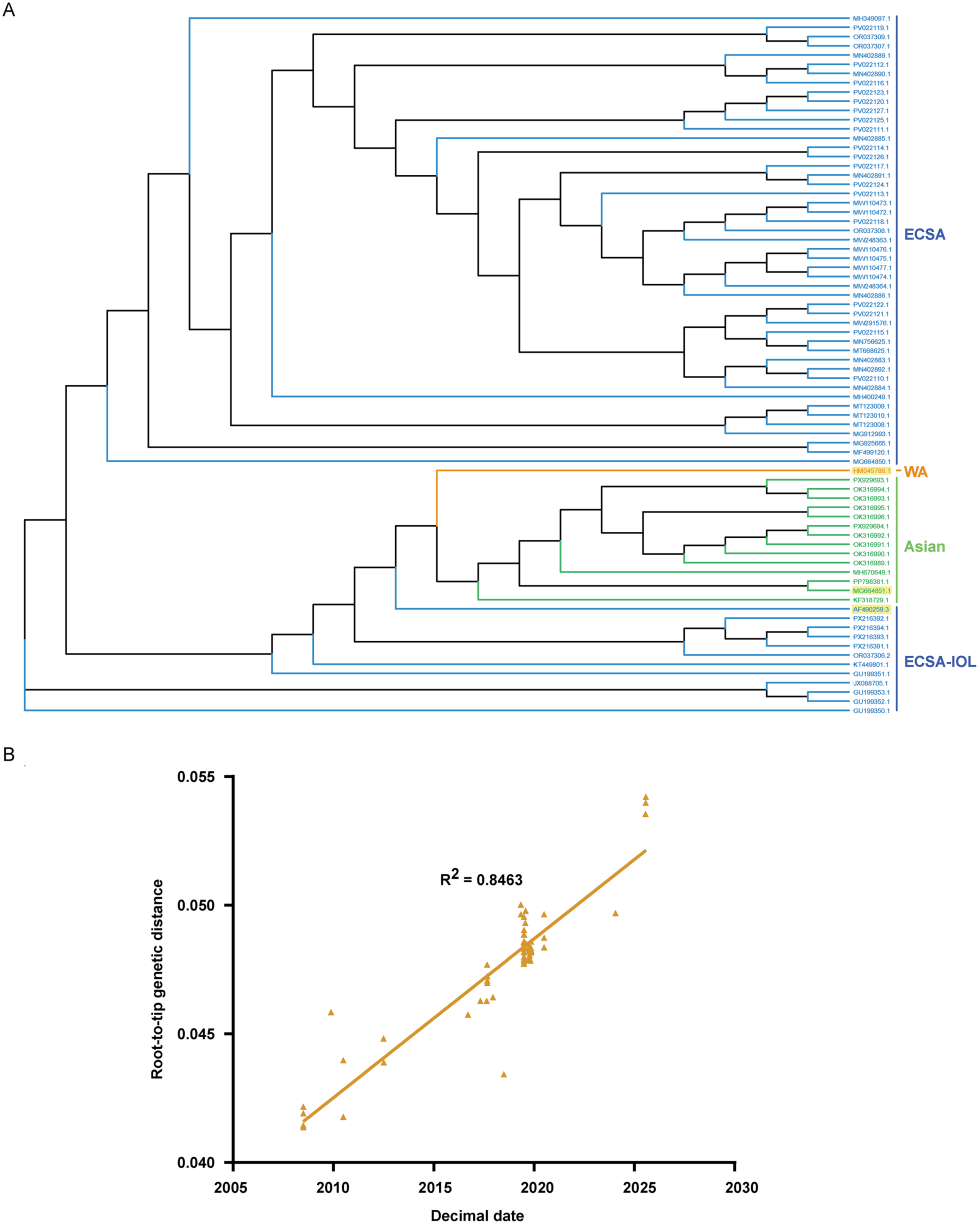
Phylogenetic analysis and divergence of CHIKV sequences from China (2006–2026). **(A)** Maximum-likelihood phylogenetic tree of CHIKV sequences originating from China. The tree includes sequences from 2006 to 2026: ECSA and ECSA-IOL (blue), Asian (green), and West African (orange). Colored blocks (yellow) denote the standard strains corresponding to each subtype. **(B)** Root-to-tip regression plot of CHIKV genomes from China. Each point represents a genome, with the X-axis showing the sampling date (in decimal years) and the Y-axis representing genetic divergence from the tree root.

The ML tree based on full-length sequences shows that all samples belong to three previously identified branches: ECSA, Asian, and WA, with no new CHIKV subtypes emerging (Figure 4A). The sequences in our dataset form a well-supported monophyletic ECSA strain, and we investigated the main mutant subtype ECSA-IOL, which is present in the sequences. During the sampling period from 2006 to 2026, CHIKV strains were found scattered across three different branches, indicating that the CHIKV identified in China during this time likely originated from multiple regions and countries. Furthermore, public data indicates that local CHIKV outbreaks in China have been extremely rare over the past two decades, suggesting that most sequences are derived from imported CHIKF cases. This indicates that imported cases are widespread, predominantly consisting of the ECSA strain, followed by the Asian strain, which aligns with our previous findings.

### 3.7 Evolutionary rate of the China imported CHIKV strains

Molecular clock analysis was conducted to estimate the evolutionary rate of the CHIKV sequences in our dataset. Root-to-tip regression analysis based on the maximum likelihood tree revealed a highly significant linear positive correlation between genetic distance and sampling time, with a strong temporal signal (R^2^ = 0.8463), indicating that sampling time accounts for 84.63% of the genetic variation among the sequences (Figure 4B). Our CHIKV sequences dataset exhibited a substitution rate of 6.0 × 10^−4^ substitutions/site/year, consistent with the range of global CHIKV evolutionary rates (Jayadas et al., 2025). The negative intercept of the regression line with the Y-axis (−1.198) suggests that the most recent common ancestor (tMRCA) of these sequences existed before the first sampling time point (2006), which aligns with the natural history of the virus.

### 3.8 Mutation analysis within the structural proteins (capsid-E1) of CHIKV

**Figure 5:**
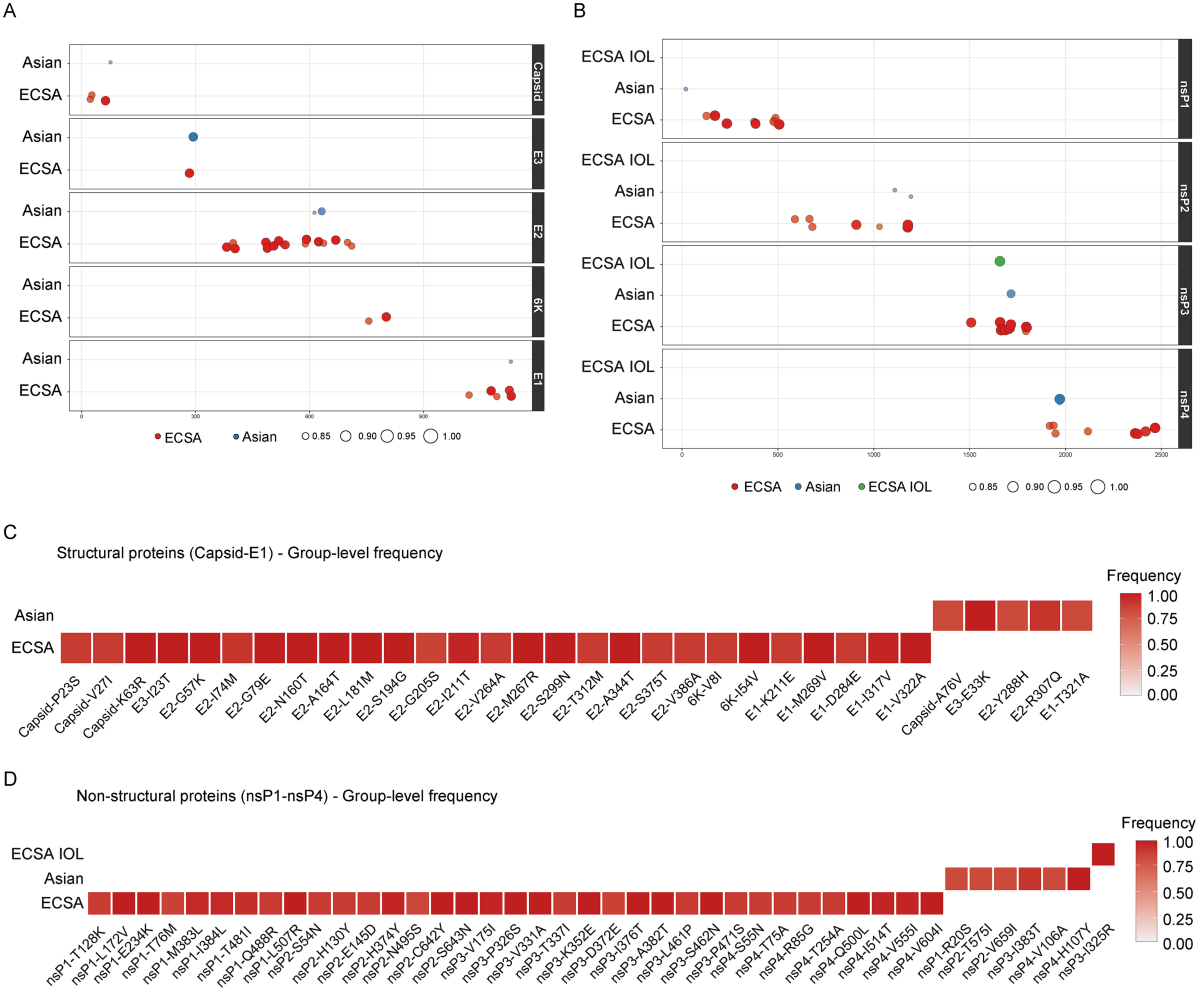
Analysis of mutations in structural and non-structural proteins. **(A)** Density of structural protein mutations in chikungunya virus strains (ECSA and Asian). **(B)** Density of non-structural protein mutations in chikungunya virus strains (ECSA, ECSA-IOL, and Asian). **(C)** Mutation frequencies at different sites of structural proteins (threshold = 0.90). **(D)** Mutation frequencies at different sites of non-structural proteins (threshold = 0.90).

Mutations that occurred in CHIKV sequences from China between 2006 and 2026 involved all structural proteins: capsid, E3, E2, 6K, and E1 (Figure 5A). These mutations were predominantly observed in the ECSA strain among the available sequences, which also had a larger number of imported cases. Among these structural proteins, the highest mutation frequency was found in E2, followed by E1. Mutations in E2 included a consistent set of 9 mutational sites (frequency = 1.00; Figure 5C). Additionally, capsid showed the K63R substitution, while E1 had the M269V and V322A consistent substitutions. In the Asian strain, no mutations at 6 K were found, with only the E33K substitution detected in E3. The ECSA IOL strain was reported in imported cases, consistent with our results showing E1-A226V (Table 3). Mutations E2-I211T, E2-V264A, and E1-K211E, reported in imported cases in China, were also confirmed in this study (Figure 5C). They exhibited relatively high mutational frequencies: E2-I211T = 0.980, E2-V264A = 0.902, and E1-K211E = 0.902. The implications of other mutation sites on CHIKV are not yet fully understood and may represent potential sites for further investigation.

**Table 3:** Mutation of structural proteins (capsid-E1).

Strain	Ref-ID	Position	Ref-AA	Mutant-AA	Frequency
ECSA	AF369024.2	23	P	S	0.902
ECSA	AF369024.2	27	V	I	0.902
ECSA	AF369024.2	63	K	R	1
ECSA	AF369024.2	73	K	R	0.7843
ECSA	AF369024.2	122	K	R	0.0784
ECSA	AF369024.2	143	T	A	0.0784
ECSA	AF369024.2	284	I	T	1
ECSA	AF369024.2	381	I	L	0.0588
ECSA	AF369024.2	382	G	K	1
ECSA	AF369024.2	399	I	M	0.9216
ECSA	AF369024.2	404	G	E	1
ECSA	AF369024.2	410	V	A	0.0784
ECSA	AF369024.2	443	S	G	0.098
ECSA	AF369024.2	474	K	R	0.0784
ECSA	AF369024.2	485	N	T	1
ECSA	AF369024.2	489	A	T	1
ECSA	AF369024.2	506	L	M	1
ECSA	AF369024.2	519	S	G	1
ECSA	AF369024.2	530	G	S	0.8824
ECSA	AF369024.2	535	L	Q	0.0784
ECSA	AF369024.2	536	I	T	0.9804
ECSA	AF369024.2	547	V	I	0.0784
ECSA	AF369024.2	552	A	V	0.0784
ECSA	AF369024.2	571	A	D	0.0784
ECSA	AF369024.2	589	V	A	0.902
ECSA	AF369024.2	592	M	R	1
ECSA	AF369024.2	607	Q	K	0.0784
ECSA	AF369024.2	624	S	N	1
ECSA	AF369024.2	637	T	M	0.902
ECSA	AF369024.2	669	A	T	1
ECSA	AF369024.2	700	S	T	0.902
ECSA	AF369024.2	711	V	A	0.902
ECSA	AF369024.2	740	I	L	0.0784
ECSA	AF369024.2	756	V	I	0.902
ECSA	AF369024.2	802	I	V	1
ECSA	AF369024.2	808	S	N	0.0784
ECSA	AF369024.2	818	N	S	0.0784
ECSA	AF369024.2	846	T	I	0.0784
ECSA	AF369024.2	1,020	K	E	0.902
ECSA	AF369024.2	1,035	A	V	0.098
ECSA	AF369024.2	1,059	S	P	0.0784
ECSA	AF369024.2	1,078	M	V	1
ECSA	AF369024.2	1,093	D	E	0.902
ECSA	AF369024.2	1,126	I	V	0.9804
ECSA	AF369024.2	1,131	V	A	1
ECSA	AF369024.2	1,133	K	R	0.0784
ECSA	AF369024.2	1,157	G	E	0.0784
ECSA	AF369024.2	1,208	V	I	0.0784
Asian	MG664851.1	76	A	V	0.8462
Asian	MG664851.1	81	M	T	0.0769
Asian	MG664851.1	294	E	K	1
Asian	MG664851.1	330	H	R	0.3077
Asian	MG664851.1	352	S	P	0.0769
Asian	MG664851.1	389	W	R	0.2308
Asian	MG664851.1	448	H	R	0.6923
Asian	MG664851.1	467	H	Y	0.0769
Asian	MG664851.1	531	S	L	0.0769
Asian	MG664851.1	535	L	S	0.0769
Asian	MG664851.1	546	K	R	0.0769
Asian	MG664851.1	558	K	E	0.7692
Asian	MG664851.1	573	S	F	0.0769
Asian	MG664851.1	613	Y	H	0.8462
Asian	MG664851.1	632	R	Q	0.9231
Asian	MG664851.1	695	A	V	0.7692
Asian	MG664851.1	696	V	L	0.0769
Asian	MG664851.1	874	T	A	0.6154
Asian	MG664851.1	944	V	I	0.5385
Asian	MG664851.1	1,005	R	K	0.0769
Asian	MG664851.1	1,100	V	A	0.2308
Asian	MG664851.1	1,130	T	A	0.8462
Asian	MG664851.1	1,138	A	V	0.1538
Asian	MG664851.1	1,142	M	V	0.0769
Asian	MG664851.1	1,206	P	L	0.1538
ECSA IOL	KT449801.1	8	T	A	0.25
ECSA IOL	KT449801.1	23	P	S	1
ECSA IOL	KT449801.1	27	V	I	0.875
ECSA IOL	KT449801.1	79	N	S	0.25
ECSA IOL	KT449801.1	303	V	I	0.125
ECSA IOL	KT449801.1	320	P	S	0.125
ECSA IOL	KT449801.1	399	M	I	0.125
ECSA IOL	KT449801.1	401	A	T	0.25
ECSA IOL	KT449801.1	503	R	H	0.125
ECSA IOL	KT449801.1	577	K	Q	0.375
ECSA IOL	KT449801.1	589	V	A	0.375
ECSA IOL	KT449801.1	638	H	Y	0.125
ECSA IOL	KT449801.1	702	I	T	0.125
ECSA IOL	KT449801.1	779	V	I	0.125
ECSA IOL	KT449801.1	870	K	Q	0.125
ECSA IOL	KT449801.1	1,020	K	E	0.375
ECSA IOL	KT449801.1	1,035	V	A	0.625
ECSA IOL	KT449801.1	1,059	S	P	0.125
ECSA IOL	KT449801.1	1,078	V	M	0.125
ECSA IOL	KT449801.1	1,125	A	V	0.125
ECSA IOL	KT449801.1	1,126	I	V	0.25

Threshold = 0.05.

### 3.9 Mutation analysis within the non-structural proteins (nsP1-nsP4) of CHIKV

Similar to the findings for structural proteins, mutations that occurred in CHIKV sequences from China between 2006 and 2026 involved all non-structural proteins (nsP1-nsP4), predominantly in the ECSA strain (Figure 5B). The majority of mutations were located in nsP3. Among the available sequences, there were seven consistent substitutions in nsP3 of the ECSA strain (Figure 5D), one of which was found in the ECSA IOL (nsP3-I325R). In addition, consistent mutations were identified in both nsP1 and nsP4, as well as C642Y and S643N in nsP2 (frequency = 1.00).

## 4 Discussion

In this study, we found that over the past 20 years, the majority of CHIKF cases in China were imported from South and Southeast Asia. The outbreak that began in Guangdong in 2025 was also triggered by imported cases from Sri Lanka (Wang et al., 2025). The peak of imported cases occurred in 2019 but then sharply declined, likely due to the strict travel restrictions imposed during the COVID-19 pandemic. After international travel resumed, the number of imported cases rebounded, culminating in a large-scale epidemic in 2025. The introduction of CHIKV into China is influenced by the diversification of importation routes, viral mutations, and the transmission vectors involved.

Imported cases of CHIKV can be categorized in two ways: (i) Cross-border transmission, facilitated by the geographical proximity along land borders, and (ii) Ports transmission, which relies on international interactions (Figure 3A). Yunnan province, for instance, has no natural barriers separating it from Myanmar and experiences frequent exchanges between the two regions. This ease of movement allows imported cases to quickly enter the country and trigger local outbreaks. In 2019, 23 cases imported from Myanmar to Yunnan province resulted in an outbreak of CHIKF in Ruili, with over 100 local cases confirmed (Liu et al., 2022). Regions such as Taiwan, Guangdong, and Hong Kong, which are located along key trade routes in China, also see high rates of importation via ports. The China Import and Export Fair, held every October in Guangdong, attracts numerous international trade personnel (Figure 3B), coinciding with the peak season for CHIKV (Figure 2D) (Huber et al., 2018). The humid, subtropical monsoon climate of the region is conducive to the breeding of *Aedes albopictus* (Qiaoli et al., 2012), further elevating the risk of local transmission. Coastal cities with numerous ports, including Zhejiang, Tianjin, and Beijing, attract international tourists and trade year-round, increasing the likelihood of CHIKV importation. The majority of imported cases are among Chinese citizens returning from South Asia and Southeast Asia, with over half (61.8%) attributed to tourism and 20.6% related to work. Notably, the large-scale transmission of CHIKV in Guangdong Province in July 2025 was linked to returning workers from Sri Lanka.

CHIKV is classified into three genotypes: the Asian genotype, WA, and ECSA (Pialoux et al., 2007). Through the construction of phylogenetic trees and molecular clocks, the imported cases of Chikungunya in China over the past 20 years have exhibited an evolutionary rate that closely mirrors that of the global Chikungunya virus, falling within the three identified types. No new strains have emerged, indicating that the imported cases are closely linked to global interactions.

New mutations frequently arise as the virus adapts to varying conditions. The research revealed that the mutation frequencies of E1 and E2 were relatively high, with a significant degree of consistent substitutions. Notably, in 2008, the E1-A226V and E2-K252Q mutations of the ECSA IOL strain were first identified in returning passengers from Malaysia to China (Zheng et al., 2010). Due to timely customs quarantine, no secondary cases occurred. Between 2017 and 2019, most imported cases in China were ECSA IOL and demonstrated multiple mutations, including E1-K211E and E2-V264A (Ho et al., 2018; Su et al., 2023), which represent adaptive mutations in the prevalent *Aedes aegypti* (Tsetsarkin et al., 2009). E2-V264A and E1-K211E exhibit a high mutational frequency, indicating that these mutations have adapted to the Chinese environment and have been preserved. The ECSA IOL with the E1-A226V mutation became widespread in 2010, causing the first cluster outbreak in Guangdong (Wu et al., 2013). In 2025, the imported cases from Sri Lanka to Guangdong Province and the subsequent widespread outbreaks were attributable to the E1-A226V mutant strain of ECSA, which also carried E2-1211T and nsP3-S259P mutations (Sun et al., 2025). The E2-I211T mutation exhibits a relatively high mutational frequency in cases of ECSA imported to China. In contrast, the nsP3-S259P mutation has a lower frequency, with a threshold of 0.05 used in Table 4 and 0.90 in Figure 5, leading to its exclusion from the results. The widespread dissemination observed in 2025 could be attributed to the accumulation of multiple superimposed mutations.

**Table 4:** Mutation of non-structural proteins (nsP1-nsP4).

Strain	Ref-ID	Position	Ref-AA	Mutant-AA	Frequency
ECSA	AF369024.2	29	P	S	0.0784
ECSA	AF369024.2	75	D	E	0.0784
ECSA	AF369024.2	95	N	S	0.0784
ECSA	AF369024.2	128	T	K	0.902
ECSA	AF369024.2	156	V	I	0.0784
ECSA	AF369024.2	172	L	V	1
ECSA	AF369024.2	234	E	K	1
ECSA	AF369024.2	290	I	V	0.1961
ECSA	AF369024.2	376	T	M	0.8824
ECSA	AF369024.2	383	M	L	0.9608
ECSA	AF369024.2	384	I	L	0.9608
ECSA	AF369024.2	481	T	I	0.9412
ECSA	AF369024.2	487	A	T	0.0784
ECSA	AF369024.2	488	Q	R	0.902
ECSA	AF369024.2	498	E	K	0.0784
ECSA	AF369024.2	507	L	R	1
ECSA	AF369024.2	569	R	C	0.0784
ECSA	AF369024.2	589	S	N	0.902
ECSA	AF369024.2	665	H	Y	0.902
ECSA	AF369024.2	680	E	D	0.902
ECSA	AF369024.2	909	H	Y	0.9804
ECSA	AF369024.2	1,030	N	S	0.8627
ECSA	AF369024.2	1,051	V	I	0.0784
ECSA	AF369024.2	1,097	P	H	0.0784
ECSA	AF369024.2	1,116	I	V	0.0784
ECSA	AF369024.2	1,139	A	V	0.0784
ECSA	AF369024.2	1,177	C	Y	1
ECSA	AF369024.2	1,178	S	N	1
ECSA	AF369024.2	1,328	A	V	0.1176
ECSA	AF369024.2	1,352	V	A	0.0784
ECSA	AF369024.2	1,455	T	I	0.0784
ECSA	AF369024.2	1,508	V	I	0.9804
ECSA	AF369024.2	1,529	A	V	0.0784
ECSA	AF369024.2	1,550	Y	H	0.7059
ECSA	AF369024.2	1,659	P	S	1
ECSA	AF369024.2	1,661	Q	P	0.098
ECSA	AF369024.2	1,664	V	A	1
ECSA	AF369024.2	1,668	S	G	0.098
ECSA	AF369024.2	1,670	T	I	0.902
ECSA	AF369024.2	1,671	T	M	0.0784
ECSA	AF369024.2	1,677	Q	R	0.0784
ECSA	AF369024.2	1,685	K	E	1
ECSA	AF369024.2	1705	D	E	0.8824
ECSA	AF369024.2	1709	I	T	1
ECSA	AF369024.2	1711	T	M	0.0784
ECSA	AF369024.2	1715	A	T	1
ECSA	AF369024.2	1732	V	I	0.0784
ECSA	AF369024.2	1743	L	Q	0.0784
ECSA	AF369024.2	1772	Q	R	0.0784
ECSA	AF369024.2	1782	M	T	0.0784
ECSA	AF369024.2	1789	S	G	0.0784
ECSA	AF369024.2	1794	L	P	0.902
ECSA	AF369024.2	1795	S	N	1
ECSA	AF369024.2	1804	P	S	0.902
ECSA	AF369024.2	1816	N	S	0.0784
ECSA	AF369024.2	1909	Q	H	0.08
ECSA	AF369024.2	1918	S	N	0.9
ECSA	AF369024.2	1938	T	A	0.9
ECSA	AF369024.2	1948	R	G	0.9
ECSA	AF369024.2	1964	T	V	0.08
ECSA	AF369024.2	2093	K	N	0.08
ECSA	AF369024.2	2,117	T	A	0.9
ECSA	AF369024.2	2,329	D	G	0.06
ECSA	AF369024.2	2,334	H	Q	0.06
ECSA	AF369024.2	2,343	A	P	0.1
ECSA	AF369024.2	2,347	A	P	0.06
ECSA	AF369024.2	2,350	M	V	0.26
ECSA	AF369024.2	2,351	N	T	0.08
ECSA	AF369024.2	2,360	V	A	0.08
ECSA	AF369024.2	2,363	Q	L	1
ECSA	AF369024.2	2,377	I	T	0.98
ECSA	AF369024.2	2,418	V	I	0.98
ECSA	AF369024.2	2,467	V	I	1
Asian	MG664851.1	20	R	S	0.8462
Asian	MG664851.1	48	I	V	0.0769
Asian	MG664851.1	55	I	T	0.0769
Asian	MG664851.1	84	M	V	0.0769
Asian	MG664851.1	121	A	E	0.0769
Asian	MG664851.1	187	A	T	0.2308
Asian	MG664851.1	230	G	R	0.7692
Asian	MG664851.1	398	C	R	0.5385
Asian	MG664851.1	737	R	T	0.5385
Asian	MG664851.1	887	P	S	0.0769
Asian	MG664851.1	987	V	A	0.0769
Asian	MG664851.1	1,021	V	I	0.0769
Asian	MG664851.1	1,110	T	I	0.8462
Asian	MG664851.1	1,115	N	Y	0.7692
Asian	MG664851.1	1,194	V	I	0.8462
Asian	MG664851.1	1,205	L	M	0.0769
Asian	MG664851.1	1,282	S	A	0.2308
Asian	MG664851.1	1,325	V	A	0.0769
Asian	MG664851.1	1,348	D	G	0.0769
Asian	MG664851.1	1,410	S	T	0.0769
Asian	MG664851.1	1,450	G	R	0.7692
Asian	MG664851.1	1,557	T	I	0.0769
Asian	MG664851.1	1,667	V	A	0.0769
Asian	MG664851.1	1,697	A	T	0.0769
Asian	MG664851.1	1716	I	T	0.9231
Asian	MG664851.1	1717	G	E	0.0769
Asian	MG664851.1	1727	M	T	0.0769
Asian	MG664851.1	1746	V	I	0.0769
Asian	MG664851.1	1770	A	T	0.0769
Asian	MG664851.1	1784	L	F	0.0769
Asian	MG664851.1	1816	D	N	0.0769
Asian	MG664851.1	1827	L	P	0.1538
Asian	MG664851.1	1912	L	H	0.7692
Asian	MG664851.1	1969	V	A	0.8462
Asian	MG664851.1	1970	H	Y	1
Asian	MG664851.1	2092	K	R	0.0769
Asian	MG664851.1	2,132	F	L	0.0769
Asian	MG664851.1	2,153	M	L	0.7692
Asian	MG664851.1	2,357	I	T	0.5385
ECSA IOL	KT449801.1	120	Q	R	0.125
ECSA IOL	KT449801.1	128	T	K	1
ECSA IOL	KT449801.1	157	H	Y	0.125
ECSA IOL	KT449801.1	221	R	S	0.125
ECSA IOL	KT449801.1	230	G	R	0.125
ECSA IOL	KT449801.1	234	E	K	1
ECSA IOL	KT449801.1	299	K	E	0.125
ECSA IOL	KT449801.1	376	T	M	1
ECSA IOL	KT449801.1	407	L	P	0.125
ECSA IOL	KT449801.1	665	H	Y	0.25
ECSA IOL	KT449801.1	1,074	L	S	0.375
ECSA IOL	KT449801.1	1,112	G	R	0.125
ECSA IOL	KT449801.1	1,167	N	S	0.125
ECSA IOL	KT449801.1	1,177	Y	C	0.125
ECSA IOL	KT449801.1	1,658	I	R	1
ECSA IOL	KT449801.1	1,688	P	L	0.125
ECSA IOL	KT449801.1	1705	D	N	0.125
ECSA IOL	KT449801.1	1727	M	I	0.125
ECSA IOL	KT449801.1	1764	R	K	0.125
ECSA IOL	KT449801.1	1945	R	S	0.125
ECSA IOL	KT449801.1	1948	R	G	0.25
ECSA IOL	KT449801.1	2044	P	S	0.125
ECSA IOL	KT449801.1	2,271	F	L	0.125
ECSA IOL	KT449801.1	2,377	T	I	0.125

Threshold = 0.05.

Adaptive mutations of CHIKV are likely to expand its transmission range and enhance its transmissibility. The E1-A226V mutation, for instance, bolsters the virus’s ability to be transmitted by *Aedes albopictus*, while the E1-K211E and E2-V264A mutations improve adaptability in *Aedes aegypti*, particularly within the genetic context of E1-226A (Khongwichit et al., 2021). Emerging mutations may also represent potential changes that impact the transmission and pathogenicity of CHIKV. As global warming continues, CHIKV is expected to expand its range from tropical regions into temperate zones (Wang and Zhang, 2025). This shift will render more areas in China suitable for the survival and reproduction of *Aedes albopictus* and *Aedes aegypti*, facilitating the spread of CHIKV and heightening the risk of local transmission. The increasing globalization and deeper international exchanges further heighten the risk of CHIKV importation. Additionally, foreign travelers may venture into inland areas under policies aimed at promoting cultural exchanges, potentially introducing CHIKV to these regions.

To assess comprehensively the trends and risk levels associated with imported CHIKV, detailed information on cases (demographic data, epidemiological information such as duration of stay abroad and virus types) and mosquito vector density is essential. Unfortunately, much of this information is not publicly available. For instance, data from Taiwan has been inaccessible due to network restrictions since 2014 (Yang et al., 2016). Based on existing statistics and literature, South Asia and Southeast Asia are identified as the main sources of imported cases, with high-risk countries like Indonesia and Myanmar noted. Moreover, the differing regional environments lead to variations in primary vectors, and the virus strains associated with imported cases often display certain tendencies. The incomplete tracking of prevalent virus strains represented by imported cases may not entirely capture the mutation dynamics of CHIKV.

To analyze CHIKV sequences, obtaining the sequence from the imported case directly is ideal. However, when that is not possible, available public reports indicate that most CHIKV outbreaks in China have been linked to imported cases, with local outbreaks occurring very rarely. Therefore, the homologous sequences of CHIKV in our dataset adequately represent the context of these imported cases. Furthermore, acquiring all possible sequences is crucial. Unfortunately, due to delays in data uploads or the absence of sequencing, the number of available sequences remains relatively small. For instance, in 2006, although the WA strain was reported, no sequences were available. This limited quantity affects the calculation of mutation frequency and may result in the omission of some mutations. Nevertheless, the temporal and geographical diversity of the obtained sequences is sufficient to accurately reflect the situation regarding imported cases.

Despite China implementing various measures to prevent CHIKF outbreaks, imported cases continue to arise annually, posing a risk of recurring epidemics. To mitigate this risk, it is crucial to quickly coordinate travel medicine and monitoring systems, such as border checks, to minimize local transmission risks. Strengthening surveillance of CHIKV mutations, developing response policies, and accelerating the approval of CHIKV vaccines, such as Ixchiq (Mullard, 2024) and Vimkunya (Tindale et al., 2025), are viable strategies. Enhancing China’s travel medical services and increasing traveler awareness are essential steps to protect the health and safety of both international travelers and local residents.

## Data Availability

The original contributions presented in the study are included in the article/supplementary material, further inquiries can be directed to the corresponding author.

## Footnotes

1
https://www.chp.gov.hk/en/index.html

2
https://www.chinacdc.cn/
